# Appraising the Methodological Quality of Sports Injury Video Analysis Studies: The QA-SIVAS Scale

**DOI:** 10.1007/s40279-023-01907-z

**Published:** 2023-08-26

**Authors:** Tim Hoenig, Lina Rahlf, Jan Wilke, Inga Krauß, Dimitris Dalos, Steffen Willwacher, Patrick Mai, Karsten Hollander, Dominik Fohrmann, Tron Krosshaug, Thomas Gronwald

**Affiliations:** 1 Department of Trauma and Orthopaedic Surgery, University Medical Center Hamburg-Eppendorf, Hamburg, Germany; 2 Department of Exercise Physiology, Institute of Sports Science, Europa-Universität Flensburg, Flensburg, Germany; 3 Department of Movement Sciences, University of Klagenfurt, Klagenfurt am Wörthersee, Austria; 4 Department of Sports Medicine, University Hospital Tuebingen, Tuebingen, Germany; 5 UKE Athleticum, Center for Sports Medicine, University Medical Center Hamburg-Eppendorf, Hamburg, Germany; 6 Institute of Interdisciplinary Exercise Science and Sports Medicine, MSH Medical School Hamburg, Hamburg, Germany; 7 Institute for Advanced Biomechanics and Motion Studies, Offenburg University of Applied Sciences, Offenburg, Germany; 8 Institute of Biomechanics and Orthopaedics, German Sport University Cologne, Cologne, Germany; 9 Department of Sports Medicine, Oslo Sports Trauma Research Center, Norwegian School of Sport Sciences, Oslo, Norway

## Abstract

**Background:**

Video analysis (VA) is commonly used in the assessment of sports injuries and has received considerable research interest. Until now, no tool has been available for the assessment of study quality. Therefore, the objective of this study was to develop and evaluate a valid instrument that reliably assesses the methodological quality of VA studies.

**Methods:**

The Quality Appraisal for Sports Injury Video Analysis Studies (QA-SIVAS) scale was developed using a modified Delphi approach including expert consensus and pilot testing. Reliability was examined through intraclass correlation coefficient (ICC_3,1_) and free-marginal kappa statistics by three independent raters. Construct validity was investigated by comparing QA-SIVAS with expert ratings by using Kendall’s tau analysis. Rating time was studied by applying the scale to 21 studies and computing the mean time for rating per study article.

**Results:**

The QA-SIVAS scale consists of an 18-item checklist addressing the study design, data source, conduct, report, and discussion of VA studies in sports injury research. Inter- and intra-rater reliability were excellent with ICCs > 0.97. Expert ratings revealed a high construct validity (0.71; *p* < 0.001). Mean rating time was 10 ± 2 min per article.

**Conclusion:**

QA-SIVAS is a reliable and valid instrument that can be easily applied to sports injury research. Future studies in the field of VA should adhere to standardized methodological criteria and strict quality guidelines.

## Key Points

**Table:** 

Multiple rounds of consensus and pilot testing led to key domains and criteria for the development of the QA-SIVAS scale as a new instrument in the quality assessment of video analysis studies.
Testing of QA-SIVAS revealed the scale to be a reliable and valid instrument that can easily be adapted into sports research.
Although designed as an assessment tool, QA-SIVAS can also act as a guide for researchers when designing and conducting studies.

## Introduction

A healthy lifestyle including physical fitness has many benefits, and participation in sports activities is increasing [9]. However, injuries are frequent among athletes of all performance levels [16, 25], and the number one cause of injury-related emergency department visits in young populations is attributed to sports participation [47]. Compared with overuse injuries [24, 35], traumatic sports injuries are less predictable and the current lack of knowledge in preventing these conditions may substantially affect long-term health [17].

Detailed analyses of how injuries happen contribute to a more comprehensive understanding of the underlying factors [3]. A holistic approach is essential and requires the assessment of both the injury situation and biomechanical patterns [3, 13, 21]. Knowledge of the cause of injury has major implications for injury prevention but is also of clinical relevance for early on-field diagnosis.

**Fig. 1:**
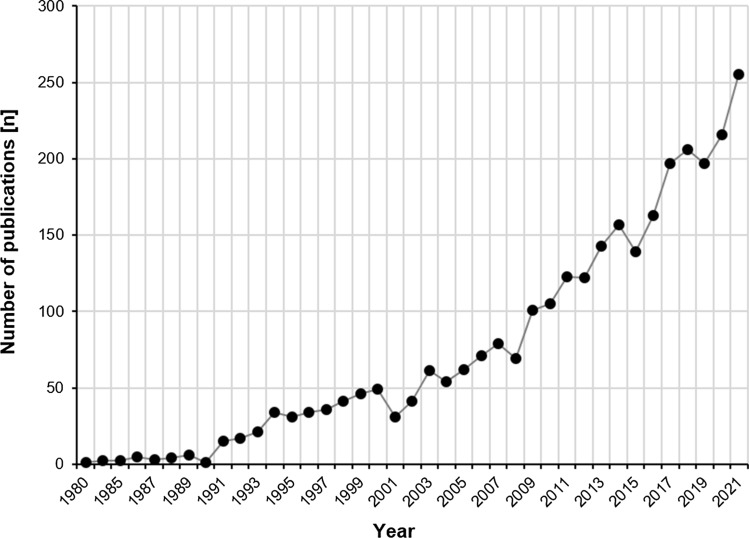
Yearly number of studies for the search term “video analysis AND injury” since 1980 (Medline, accessed via PubMed on 21 November 2022)

Video analysis (VA) has been an increasingly used tool to investigate sports injuries over the last 40 years (Fig. 1). It has gained high popularity in recent years, and there is widespread agreement regarding its scientific and clinical value to the sports medicine community [13, 21]. Among others, reports have been published describing situations, mechanisms and biomechanics of injury to the anterior cruciate ligament [13], Achilles tendon [14, 26], adductor longus muscle [43], and hamstring muscles [21]. However, to date, a lack of standardized methods is seen as a major limitation of VA studies [1]. Quality assessment tools, methodological gold standards, and consensus on terminology may increase consistency and allow a better comparison between studies. Most likely, with growing interest in the methodological framework of VA, systematic reviews will become available. A systematic appraisal of the literature provides the highest level of evidence, but can only be as good as the studies included [23]. Consequently, quality and risk of bias assessments of studies become necessary [23]. However, to date, no instruments exist for assessing study quality in video-based sports injury analysis. Thus, the objective was to develop and evaluate a tool that helps to assess the methodological quality of VA studies for the purpose of enhanced sports injury investigation.

## Materials and Methods

The present study took place between January and December 2022 in three phases: (a) development of the QA-SIVAS (Quality Appraisal for Sports Injury Video Analysis Studies) scale; (b) evaluation of its reliability and within-item agreement, and (c) assessment of its construct validity and feasibility. Ethical approval was not required as this study did not involve any patient data. All expert panelists provided written consent to participate.

### Scale Development

The QA-SIVAS scale was developed using a modified Delphi approach including expert consensus and pilot testing [47].

At first, experts in the field of sports science and sports medicine (TG, TH, LR, KH, DF, SW, PM, JW, and TK) independently listed criteria they considered important when assessing sports injuries by using VA. One reviewer (TG) summarized the group’s responses according to frequency and topic. The categorized criteria were then sent back to the experts. After comparing their submissions with those of the other reviewers, each reviewer was allowed to comment on other statements and to revise their own submissions. Subsequently, the criteria were again categorized by the same reviewer (TG). A face-to-face panel discussion with all contributing experts involved was then held, and this led to the development of the first version of QA-SIVAS. Five raters (TG, TH, LR, DF, SW) compared the scale against five randomly selected VA studies out of a pool of 21 included VA studies of anterior cruciate ligament (ACL) injuries from a recent systematic review project (PROSPERO registration: CRD42022337340). The preliminary testing was used to further adjust the rating criteria and instructions.

### Reliability

To estimate inter- and intra-rater reliability, three reviewers (TG, LR, TH) independently assessed the risk of bias of all 21 VA studies from the aforementioned recent systematic review project (PROSPERO registration: CRD42022337340) by applying the QA-SIVAS scale. The rating was repeated after 3 weeks. The reliability of the main outcome, the total QA-SIVAS score [%], was estimated by using the Intraclass Correlation Coefficient (ICC_3,1_). Results were interpreted as ‘poor’ (ICC < 0.4), ‘fair to good’ (0.4–0.75), and ‘excellent’ (> 0.75). Agreement of each QA-SIVAS item was estimated using free-marginal kappa statistics by Brennan and Prediger [39], which minimizes prevalence-related biases. Interpretation of the results was carried out based on Landis and Koch (1977): *k* < 0 (‘poor’); *k* = 0.01–0.20 (‘slight’); *k* = 0.21–0.40 (‘fair’); k = 0.41–0.60 (‘moderate’); *k* = 0.61–0.80 (‘substantial’); *k* = 0.81–1.00 (‘almost perfect’).

### Construct Validity

To evaluate construct validity, an approach similar to Jadad et al. [27] and Wilke et al. [47] was used. All 21 studies were assigned to two field-related experts, an orthopedist and sports physician (DD) and an exercise scientist and physiotherapist (IK). They independently rated each study’s quality with 1–4 points (1: poor; 2: poor to moderate; 3: moderate to good; 4: excellent). The mean ratings were correlated to the scores obtained with the QA-SIVAS scale (TG, LR, TH) using Kendall’s tau analysis.

### Rating Time

Rating time was evaluated as the required mean time for the rating of one study. All three reviewers involved in the reliability study (TG, LR, TH) recorded the time (min:sec per study article) needed to evaluate the study quality for each article. Means were calculated for all three reviewers.

Statistical calculations were performed using SPSS version 27.0 (SPSS, Chicago, IL, USA). *p*-values < 0.05 were considered significant.

## Results

The final QA-SIVAS scale consists of an 18-item checklist addressing the study design, data source, conduction, reporting, and discussion of VA studies in sports injury assessment. Each item is to be answered with either 0 (no/not stated) or 1 (yes/present) point. The maximum score is 18. The quality rating is expressed as a percentage value (reached score/maximum score [%]). Detailed information about the components and scoring guidelines is displayed in Table 1.

**Table 1:** Items and scoring criteria of the QA-SIVAS scale

No.	Item	When to score ‘yes ‘	Purpose
1	Objective stated	The study’s aims **OR** hypotheses are clearly stated	To indicate the overarching purpose of the study
2	A representative sample was chosen	The study considers a homogenous group of athletes with systematic registration of injuries; the number of injuries investigated and video recordings accessible is stated Example: Professional female football players (homogenous group of athletes) with 20 anterior cruciate ligament ruptures (systematic registration of injuries, number of injuries investigated), ten out of which were recorded on video (number of video recordings accessible)	To ensure reliable data acquisition
3	Information about sample is included	Characteristics of the study population are included (at least age, sex, sport, performance level, and sample size are stated)	To support interpretation and differentiation of the results
4	Information about video source and quality of the footage are included	Source (e.g., television or private recording, video platform) **AND** quality of video footage are described (all of the following are described: sampling frequency, quality of resolution and number and type of camera views); if mixed datasets are used, minimum and maximum values of the features are reported	To enhance objectivity of the observations
5	Applied methods are described comprehensively	A clearly structured, detailed outline of the study protocol **AND** the process of video analysis is stated	To provide a basis for comparisons with similar studies
6	A systematic approach to video analysis was chosen	A systematic approach to video analysis is used (e.g., checklist, observation form) **AND** clearly defined observation criteria are used (e.g., definition of injury mechanism and/or pattern)	To enhance objectivity of the observations
7	Medical report information is included	Study included medical report information (e.g., confirmation of diagnosis, injury location, type, diagnosis) either directly (extraction from medical reports) or through communication with medical professionals	To support interpretation and differentiation of the results
8	Background/expertise of rater(s) is stated	Relevant occupation (e.g., physician, sports scientist, physiotherapist) **AND** experience (or training in video rating) of the video rater(s) in the specific domain are reported	To evaluate the expertise of the rater
9	Findings are observed by more than one researcher	It is clearly stated that two or more raters independently made the observations	To enhance reliability of the observations
10	A control group is included	A control group with similar situations and/or biomechanics not resulting in injury is included (e.g., landing with a straight leg)	To enable the derivation of causal relationships and to avoid misinterpretation of findings
11	A quantitative biomechanical analysis was conducted using validated methods	Quantitative descriptions of biomechanics are included (e.g., joint angles and/or kinematics) **AND** have been created using validated methods (validity and reliability of the methods is reported in the methods section)	To enhance understanding of the findings
12	The main results of the study are clearly described	Results are described with a clear structure **AND** figures or tables are included	To avoid misleading interpretation of results
13	Absolute numbers or proportions of injury cases (for each/the main outcome) are reported	Absolute numbers **OR** proportions (percentage) of injury cases are included, mechanisms (e.g., non-contact vs contact) and/or pattern (e.g., knee valgus pattern vs hyperextension pattern) are reported	To allow interpretations about the frequency of a finding
14	Details about the injury context are included	Information about the injury context/situation (e.g., player action, match or training injuries, time in game, hours of exposure during game/training) are included	To enhance understanding of the findings
15	Example screenshots/video frames are included	Example screenshots/video frames of the observed injury-specific outcomes of interest are included (e.g., injury mechanisms and/or pattern)	To overcome communication barriers and to communicate research with a broad readership
16	Findings are discussed within the context of the current evidence	Other relevant trials relating to the field of study are stated and discussed	To point out the studies' contribution to what was already known
17	Clinical/practical implications of the results are discussed	Information on how to use study findings in clinical/sports practice are discussed	To help the transfer of findings into practice
18	Limitations of the study are addressed	Weaknesses **OR** methodological shortcomings are reported	To avoid misinterpretation of findings and to identify the need for future studies

### Reliability

The analysis of raters’ agreement with regard to the total score (%) revealed an ICC of 0.98, interpreted as ‘excellent’ (95% confidence interval [CI] 0.96–0.99; *p* < 0.001). Repeated scoring showed excellent intra-rater reliability for all three raters: ICC rater 1 = 0.99 (95% CI 0.99–0.99; *p* < 0.001); ICC rater 2 = 0.99 (95% CI 0.98–0.99; *p* < 0.001), and ICC rater 3 = 0.99 (95% CI 0.98–0.99; *p* < 0.001).

Free marginal kappa values of the individual items ranged between 0.68 and 1.00 (substantial to almost perfect) with an exact agreement of 84–100% (Table 2).

**Table 2:** Reliability and percent agreement of individual QA-SIVAS items

No.	QA-SIVAS scale item	Base rate	Free-marginal kappa	Kappa (95% CI)	Percent exact agreement
1	Objective stated	100	1.00	1.00–1.00	100
2	A representative sample was chosen	40	0.68	0.42–0.93	84
3	Information about sample is included	0	1.00	1.00–1.00	100
4	Information about video source and quality of the footage are included	13	0.68	0.43–0.93	84
5	Applied methods are described comprehensively	95	1.00	1.00–1.00	100
6	A systematic approach to video analysis was chosen	94	0.94	0.81–1.00	96
7	Medical report information is included	29	1.00	1.00–1.00	100
8	Background/expertise of rater(s) is stated	49	0.81	0.61–1.00	90
9	Findings are observed by more than one researcher	70	0.94	0.81–1.00	96
10	A control group is included	14	1.00	1.00–1.00	100
11	A quantitative biomechanical analysis was conducted using validated methods	13	0.86	0.67–1.00	92
12	The main results of the study are clearly described	95	0.87	0.70–1.00	93
13	Absolute numbers or proportions of injury cases (for each/the main outcome) are reported	95	0.87	0.70–1.00	93
14	Details about the injury context are included	92	0.94	0.81–1.00	96
15	Example screenshots/video frames are included	76	1.00	1.00–1.00	100
16	Findings are discussed within the context of the current evidence	100	1.00	1.00–1.00	100
17	Clinical/practical implications of the results are discussed	90	0.81	0.61–1.00	90
18	Limitations of the study are addressed	100	1.00	1.00–1.00	100

Base rate refers to the percentage of ratings that were answered with yes/present out of 63 ratings per scale item (21 studies, 3 reviewers)

### Construct Validity

The analysis of construct validity with two experts in the field revealed a strong association of the results obtained by means of the QA-SIVAS scale and the expert ratings (Kendall’s tau *B* = 0.71, *p* < 0.001; see Table 3).

**Table 3:** Ratings for the validity analysis made by two experts in the field (point scale: 1–4) and the corresponding QA-SIVAS scores (point scale: 0–18) for the included 21 studies

Study	Expert 1	Expert 2	Mean	QA-SIVAS score (%)
Belcher et al. (2022) [4]	3	3	3	12 (67)
Boden et al. (2000) [5]	1	1	1	6 (33)
Boden et al. (2009) [6]	2	2	2	9 (50)
Brophy et al. (2015) [7]	2	2	2	11 (61)
Cochrane et al. (2007) [10]	3	3	3	11 (61)
De Carli et al. (2022) [12]	1	3	2	10 (56)
Della Villa et al. (2020) [13]	4	4	4	14 (78)
Della Villa et al. (2021) [15]	4	4	4	14 (78)
Grassi et al. (2017) [19]	3	3	3	11 (61)
Grassi et al. (2020) [20]	3	3	3	11 (61)
Johnston et al. (2018) [28]	3	3	3	12 (67)
Koga et al. (2010) [30]	3	4	3.5	12 (67)
Koga et al. (2017) [29]	4	4	4	11 (61)
Krosshaug et al. (2007) [31]	3	3	3	13 (72)
Lucarno et al. (2021) [32]	4	4	4	14 (78)
Montgomery et al. (2018) [36]	4	4	4	14 (78)
Olsen et al. (2004) [38]	4	4	4	13 (72)
Rolley et al. (2023) [40]	3	3	3	10 (56)
Sheehan et al. (2012) [44]	2	3	2.5	10 (56)
Stuelcken et al. (2016) [45]	3	3	3	13 (72)
Waldén et al. (2015) [46]	4	4	4	15 (83)

### Rating Time

The mean rating time among all three reviewers was 10:07 ± 02:27 min:sec per article.

## Discussion

A state-of-the-art method was applied to develop the QA-SIVAS scale. The tool covers 18 distinct items. Scores of each item can be used cumulatively for an overall judgment of a study’s quality. Inter- and intra-rater reliability, construct validity and user rating time were excellent. Consequently, the QA-SIVAS scale can be used by researchers for judging the quality of studies using video analysis of injuries in sports.

To our knowledge, QA-SIVAS is the first scale that addresses the quality of studies using video-based assessment of sports injuries. As no assessment instrument has been available hitherto, the scale was developed based on the consensus of an interdisciplinary multi-center reviewer team with field-specific knowledge. The final version was implemented in a systematic review covering 21 studies that have investigated ACL injuries in sports (PROSPERO registration: CRD42022337340).

The intra- and inter-rater reliability were excellent, indicating that the QA-SIVAS scale can be reliably used. This finding is comparable to the risk of bias scales from other medical fields. For instance, the inter-rater reliability of the PEDro scale, a commonly used tool in the assessment of clinical trials, ranges between 0.56 and 0.91 [18, 34]. The excellent inter-rater agreement of the QA-SIVAS scale is particularly notable given the multidisciplinary reviewer team of medical doctors, biomechanists, and sports scientists. Potentially, a background in sports injury research may be necessary, as this was a given prerequisite for all reviewers involved in the development of QA-SIVAS. However, the involvement of coaches and players in the research team has clear benefits, and is recommended [42]. Construct validity of the scale can be interpreted as high based on the correlation analysis revealing a Kendall’s tau *B*-value of 0.71. All assessments were done in a reasonable amount of time (average duration of around 10 min per article). This is comparable to other quality appraisal instruments and supports its feasibility [37, 47]. The rating time may be even less than 10 min if the raters know the studies beforehand (e.g., from performing a systematic literature search).

Our results from the evaluation process give some guidance on how to interpret QA-SIVAS. Most studies included through the systematic search on ACL injuries scored between 60 and 80%. No study achieved maximal scoring. Only a few studies scored < 60%. One study scored > 80%. However, there was only one item (item 3; Characteristics of the study population are included) that was not met by any of the included studies. Although no study has met the criteria of this item (by not reporting the age of the study population in the majority of studies), item 3 was still considered indispensable due to its clinical implication for injury prevention. It may thus be proposed to use the following interpretation when judging study quality: < 60%: low quality; 60–70%: moderate quality; 71–80%: good quality, 81–100%: high quality. Accordingly, five studies were of low quality, eight studies of moderate quality, seven studies of good quality, and one study of high quality.

For studies already completed, the QA-SIVAS scale may help to highlight methodological concerns. The QA-SIVAS scale can thus be used for assessing multiple studies that are included in a systematic review. High-quality systematic reviews are of great importance [41], and can support clinical decision making in sports [2]. However, shortcomings in the quality of included studies are of major concern. Quality assessment of studies is a challenging but indispensable task in order to draw valid conclusions based on the available evidence on a given topic [48]. QA-SIVAS can be used when conducting systematic reviews, and may also help researchers assess the quality of individual studies.

Although designed as an assessment tool, QA-SIVAS can also be a guidance for researchers. To date, only few recommendations on how to standardize VA have been published. These recommendations were specific to sports or injuries such as concussion, rugby or netball [11, 22, 33]. For example, in future VA research, QA-SIVAS may improve study design when used as a checklist in the planning of new studies [48]. By developing the QA-SIVAS scale, it was the firm intention of our group to extend the principles of evidence-based medicine into the field of video-based sports injury assessment. Future studies in the field should adhere to the QA-SIVAS criteria. In the planning of future studies, items 2 and 11 should be given special attention to allow for a homogenous and standardized methodological approach. When reporting study results, particular attention may be given to items 3, 4, and 8 of the QA-SIVAS scale. These data can easily be included when reporting study results. However, only a minority of the studies assessed fulfilled these items.

### Strength and Limitations

To the best of our knowledge, the QA-SIVAS scale is the first specific tool to assess the quality of studies using VA of injuries in sports. It was developed and refined based on multiple rounds of consensus. However, reliability and validity testing was applied to anterior cruciate ligament ruptures without considering other injury mechanisms. Further, the independence of reviewers is not granted as some of the authors have previously worked together on research projects [8]. For this reason, it was a strategic goal to include multidisciplinary reviewers from multiple centers. Nevertheless, the practical implementation of QA-SIVAS is pending. As with other tools, the significance of QA-SIVAS will depend on feedback from the research community and updates may be required in future years.

## Conclusion

We identified key domains and criteria for the evaluation and development of studies in the field of video-based assessment of sports injuries. By using multiple rounds of consensus and pilot testing, the QA-SIVAS scale was developed as a new instrument in the quality assessment of studies in this field. We have provided evidence that QA-SIVAS is reliable, valid, and can be easily adapted into sports research. Future studies in the field of video-based analysis of sports injuries should adhere to standardized methodological criteria and strict quality guidelines.

## Declarations

### Availability of Data and Material

All data generated or analysed during this study are included in the published article.
